# Prognostic impact of activin subunit inhibin beta A in gastric and esophageal adenocarcinomas

**DOI:** 10.1186/s12885-022-10016-5

**Published:** 2022-09-05

**Authors:** J. J. Staudacher, Alexander Arnold, A. A. Kühl, M. Pötzsch, S. Daum, M. Winterfeld, E. Berg, M. Hummel, B. Rau, U. Stein, C. Treese

**Affiliations:** 1 Medical Department, Division of Gastroenterology, Infectious Diseases and Rheumatology, Charité - Universitätsmedizin Berlin, Campus Benjamin Franklin, Berlin, Germany; 2 Berlin Institute of Health at Charité Universitätsmedizin Berlin, Charitéplatz1, 10117 Berlin, Germany; 3 Institute of Pathology, Charité - Universitätsmedizin Berlin, Berlin, Germany; 4 Charité – Universitätsmedizin Berlin, corporate member of Freie Universität Berlin and Humboldt Universität zu Berlin, iPATH.Berlin, Campus Benjamin Franklin, Berlin, Germany; 5 Department of Surgery, Campus Virchow-Klinikum and Campus Mitte, Charité - Universitätsmedizin, Berlin, Germany; 6 Experimental and Clinical Research Center, Charité – Universitätsmedizin and Max-Delbrück-Center for Molecular Medicine in the Helmholtz Association, Berlin, Germany; 7 German Cancer Consortium (DKTK), Heidelberg, Germany

**Keywords:** Gastric adenocarcinoma, Esophageal adenocarcinoma, Activin, INHBA, TGF-β superfamily

## Abstract

**Purpose:**

Adenocarcinomas of the esophagus (AEG) and stomach (AS) are among the most common cancers worldwide. Novel markers for risk stratification and guiding treatment are strongly needed. Activin is a multi-functional cytokine with context specific pro- and anti-tumorigenic effects. We aimed to investigate the prognostic role of activin tumor protein expression in AEG/ASs.

**Methods:**

Tissue from a retrospective cohort of 277 patients with AEG/AS treated primarily by surgery at the Charité - Universitätsmedizin Berlin was collected and analyzed by immunohistochemistry using a specific antibody to the activin homodimer inhibin beta A. Additionally, we evaluated T-cell infiltration and PD1 expression as well as expression of PD-L1 by immunohistochemistry as possible confounding factors. Clinico-pathologic data were collected and correlated with activin protein expression.

**Results:**

Out of 277 tumor samples, 72 (26.0%) exhibited high activin subunit inhibin beta A protein expression. Higher expression was correlated with lower Union for International Cancer Control (UICC) stage and longer overall survival. Interestingly, activin subunit expression correlated with CD4^+^ T-cell infiltration, and the correlation with higher overall survival was exclusively seen in tumors with high CD4^+^ T-cell infiltration, pointing towards a role of activin in the tumor immune response in AEG/ASs.

**Conclusion:**

In our cohort of AEG/AS, higher activin subunit levels were correlated with longer overall survival, an effect exclusively seen in tumors with high CD4^+^ cell infiltration. Further mechanistic research is warranted discerning the exact effect of this context specific cytokine.

**Supplementary Information:**

The online version contains supplementary material available at 10.1186/s12885-022-10016-5.

## Introduction

Adenocarcinomas of the esophagus (AEG) and stomach (AS) are among the most common cancers worldwide [1]. Despite slowly falling incidence rates of AS in developed countries most probably due to lower *H. pylori* infection rates [2], AEGs are diagnosed more frequently, coinciding with an increase in Barrett’s esophagus [3]. Albeit therapeutic advances, prognosis remains dire especially in a metastasized setting with 5-year survival rates around 5 % [1]. Novel markers for risk stratification and guiding treatment are strongly needed.

Activin, a transforming growth factor-beta (TGF-β) superfamily member is a multifunctional cytokine with a well-recognized role in the tumorigenesis of gastrointestinal tumors [4]. Initially described as an upstream effector of follicle stimulating hormone [5], additional roles of activin were shown in contexts as inflammation [6–9], cancerogenesis [4, 10, 11] and cancer cachexia [12]. The pleiotropic and context-specific effects of the activin pathway can be partly understood through the complexity of activin signaling. Activin ligand consist of two Inhibin beta subunits with several isoforms, the most abundant and best described being activin A, consisting of two Inhibin beta A subunits. Besides the homodimer, a heterodimer of Inhibin beta A and Inhibin alpha exists. For the sake of readability, activin will be used in this manuscript in lieu of activin homodimer subunit beta A. It binds to its type 2 receptor which in turn dimerizes and phosphorylates the activin type 1 receptors [13], leading to activation of socalled canonical SMAD-dependent and numerous non-canonical pathways, including the PI3K, MAP/ERK and NFkappaB pathways [14, 15].

In tumorigenesis, pro and anti-oncogenic functions are documented. In early stage colorectal cancer, activin acts similarly to TGF-β growth-inhibiting through SMAD-dependent signaling. In later stages, activin acts primarily pro-metastatic through non-canonical pathways [16]. Additionally, first reports on a role of activin in the tumor immune response are emerging. The literature describes pro- and anti-inflammatory effects of activin, which are also characteristic for other TGF-beta superfamily members [17]. Initially best characterized for strictly pro-inflammatory effects on the innate immune system, especially neutrophils [18], new data point to an immune modulating function of activin signaling with context specific pro- and anti-inflammatory effects. Specifically, activin was shown to induce CD4^+^ regulatory T-cells both in the context of inflammatory conditions such as allergic airway disease [19] and in the context of cancer. Strikingly, both pro- and anti-tumorigenic effects of activin through CD4 cells were described in the literature. In the context of breast and skin cancer, activin was shown to facilitate immune evasion through its action on CD4^+^ regulatory T-cells [20]. Recently, activin was shown to drive differentiation of CD4^+^ cells into Th17 cells [21].

Data on activins role in the context of AEG/AS are sparse. Multiple investigators have shown a growth-inhibitory and pro-apoptotic function of activin in gastric cancer cell lines [22, 23] . Additionally, and consistent with activins dual role in other tumors such as colorectal cancer, mRNA silencing of activin leads to reduced cell migration and invasion in vitro [24]. Data from two Japanese cohorts demonstrate higher activin mRNA-expression correlating with worse prognosis and shorter overall survival in patients after primary resection [25] or after adjuvant chemotherapy and resection [26], respectively. Additionally, in a Chinese cohort from patients with AEG/AS, Wang et al. reported a correlation between protein expression of the activin subunit inhibin beta A and shorter overall survival [27]. As the five-year survival rate in this cohort was around 80%, the transferability of these results is questionable.

Overall, the in vitro data show an anti-oncogenic effect, but the cohort data show a positive correlation with poor survival. Despite its known immune modulating effects [28] correlation of activin expression and tumor infiltrating lymphocytes in AEG/ASs are missing.

The aim of this study was to investigate the prognostic role of activin subunit inhibin beta A in a large Caucasian AEG/AS cohort and to test a potential correlation of activin expression and level of lymphocyte infiltration.

## Material & Methods

### Cohort

Clinical data from patients with AGE/AS of all tumor stages, primarily treated by surgery between 1992 and 2004 at the Charité - Universitätsmedizin Berlin, were collected retrospectively. From 2004 onwards, patients were treated with (neo)-adjuvant chemotherapy at our institution. As the aim of this study was to investigate the prognostic impact of activing subunit inhibin beta A and for the sake of comparability, we specifically chose a cohort exclusively treated by surgery. The mean follow-up was 113.6 months (95% CI: 103.9–123.2). Overall survival was defined as time from diagnosis to death or last follow-up. Disease-specific survival was defined as time from diagnosis to tumor-related death or last follow-up. The data including patient characteristics and follow-up information were retrieved from the patient management software (SAP®) and the regional population-based cancer registry (“Gemeinsames Krebsregister”) and are summarized in Table 1. All patients included in this study gave their informed consent prior to their inclusion. This study was approved by the Institutional Review Board of the Charité (EA4/115/10).

**Table 1:** Patient characteristics of the analyzed patient cohort and distribution of Activin high and low expressing primary tumors. Significance calculated by X^2^-Test

	All		Activin				
	Total		low.		high.		p
	n	(%)	n	(%)	n	(%)	
Gender							
Female	100	(36.1)	78	78.0	22	22.0	0.298
Male	177	(63.9)	128	72.3	49	27.7	
Age Group							
< 65 years	156	(56.3)	110	70.5	46	29.5	0.095
> = 65 years	121	(43.7)	96	79.3	25	20.7	
Localization							
Gastric Cancer	230	(83.0)	171	74.3	59	25.7	0.986
AEG	47	(17.0)	35	74.5	12	25.5	
Tumor stage							
T1	38	(13.7)	21	55.3	17	44.7	**0.041**
T2	105	(37.9)	78	74.3	27	25.7	
T3	102	(36.8)	80	78.4	22	21.6	
T4	31	(11.2)	26	83.9	5	16.1	
unspecified	1	(0.4)	0	0.0	1	100.0	
Node Stage							
N0	71	(25.6)	45	63.4	26	36.6	**0.014**
N+	206	(74.4)	161	78.2	45	21.8	
Distante Metastasis							
M0	196	(70.8)	136	69.4	60	30.6	**0.003**
M1	81	(29.2)	70	86.4	11	13.6	
Lymphatic vessel invasion							
L0	92	(33.2)	63	68.5	29	31.5	0.137
L1	165	(59.6)	127	77.0	38	23.0	
unspecified	20	(7.2)	–		–		
Vein invasion							
V0	164	(59.2)	116	70.7	48	29.3	0.107
V1	90	(32.5)	72	80.0	18	20.0	
unspecified	23	(8.3)	–		–		
Grading							
G1	1	(0.4)	0	0.0	1	100.0	**0.35**
G2	73	(26.4)	48	64.9	26	35.1	
G3	200	(72.2)	157	78.5	43	21.5	
unspecified	3	(1.1)					
Lauren Classification							
Intestinal	98	(35.4)	65	66.3	33	33.7	0.114
Diffuse	139	(50.2)	108	77.7	31	22.3	
Mixed	37	(13.4)	31	83.8	6	16.2	
unspecified	3	(1.1)					
Ming Classification							
expansive	109	(39.4)	72	66.1	37	33.9	**0.034**
infiltrative	165	(59.6)	132	80.0	33	20.0	
unspecified	3	(1.1)					
MMR							
proficient	240	(86.6)	178	74.2	62	25.8	0.997
deficient	31	(11.2)	23	74.2	8	25.8	
unspecified	6	(2.2)	–	–	–	–	
CD3							
low	137	(49.5)	105	76.6	32	23.4	0.691
high	118	(42.6)	85	72.0	33	28.0	
unspecified	22	(7.9)	–	–	–	–	
CD4							
low	147	(60.0)	120	81.6	27	18.4	**0.009**
high	98	(35.4)	63	64.3	35	35.7	
unspecified	32	(11.6)	–	–	–	–	
CD8							
low	134	(48.4)	106	79.1	28	20.9	0.217
high	120	(43.3)	84	70.0	36	30.0	
unspecified	23	(8.3)	–	–	–	–	
PD-L1 in lymphocytes							
negative	182	(65.7)	141	77.5	41	22.5	0.065
positive	81	(29.2)	54	66.7	27	33.3	
unspecified	11	(4.0)	–	–	–	–	
PD-L1 in tumor cells							
negative	245	(88.4)	185	75.5	60	24.5	0.062
positive	18	(6.5)	10	55.6	8	44.4	
unspecified	11	(4.0)	–	–	–	–	
PD-1 in lymphocytes							
negative	112	(40.4)	90	80.4	22	19.6	0.051
Positive	145	(52.3)	101	69.7	44	30.3	
unspecified	20	(7.2)	–	–	–	–	
PD-1 in tumor cells							
negative	259	(93.5)	192	74.1	67	25.9	**–**
positive	0	(0.0)	0	0	0	0	
unspecified	18	(6.5)	–	–	–	–	

### Tissue samples

Tissue samples were collected from the archive of the Institute of Pathology, Charité - Universitätsmedizin Berlin. Paraffin embedded tumor samples (*n* = 277) were available from surgically treated chemotherapy-naive patients. All samples were reevaluated according to histological diagnosis, tumor stage and grade, and classified by the histological architecture of AGE/S carcinoma using Lauren’s and Ming classification by a specialist for gastrointestinal pathology (M.W.). Data concerning tumor size, depth of invasion, and tumor invasion of veins or lymphatic vessels were retrieved from the Charité - Universitätsmedizin Berlin patient management software. Staging was done following UICC TNM staging classification 7th Edition.

Tissue samples were screened in a HE-stained section for representative areas of solid tumors. Two 1 mm-diameter tissue cores were punched out from each of the 277 available cases and were transferred to a recipient paraffin block. After re-melting, sections (4 μm thick) were consecutively cut from each tissue microarray block (TMA). HE staining was TMA sections for reconfirmation of content of tumor and non-tumor tissue in each core.

Immunohistochemical analysis of expression of CD3^+^, CD4^+^ and CD8^+^, as well as PD-1 and PD-L1 was performed on consecutive TMA sections using a specific monoclonal antibody as previously described. Correlation of prognosis with these factors in this cohort as well as representative images of the stainings were previously published [29].

**Fig. 1:**
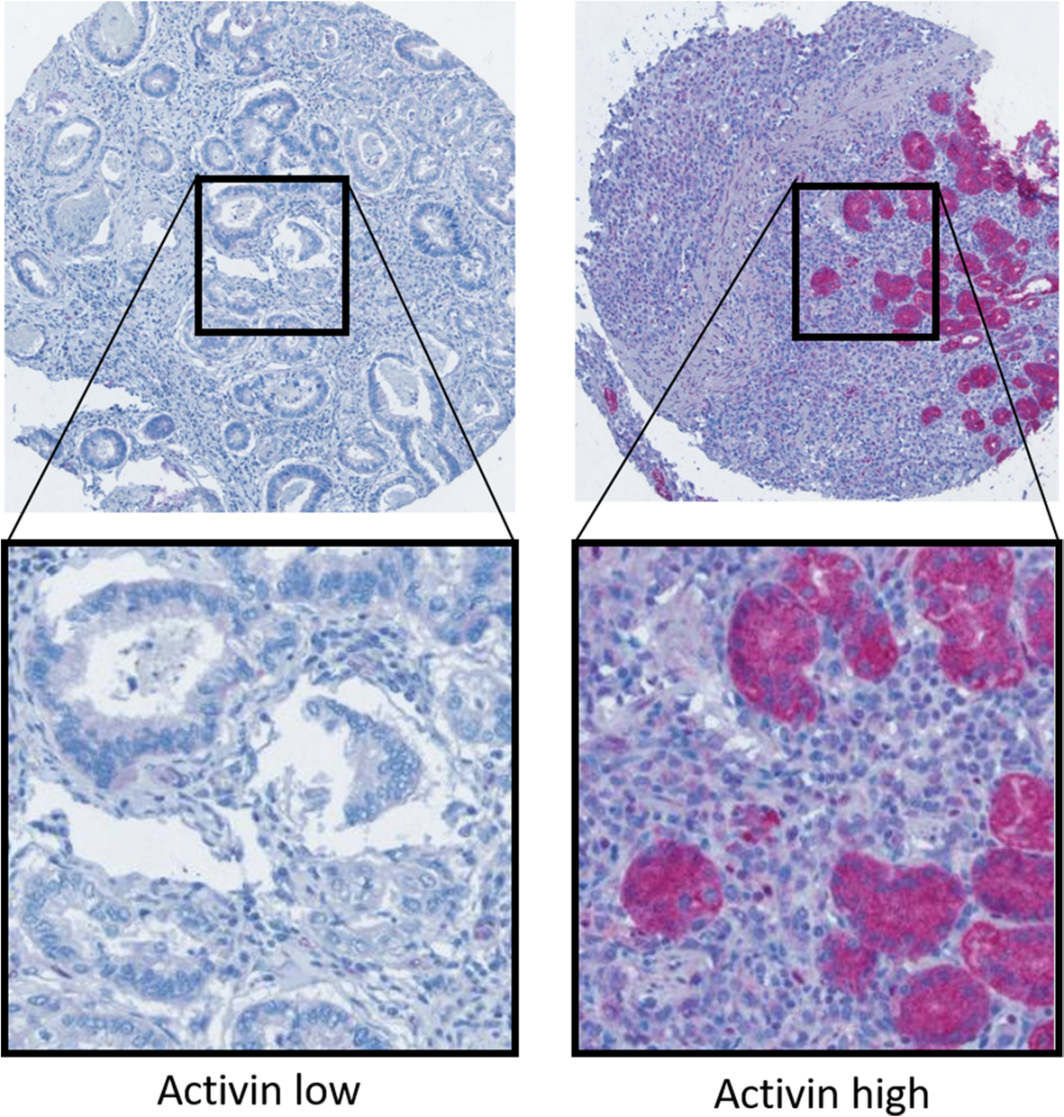
Representative activin a homodimer subunit staging 18.2 IHC Staining of TMA cores. **A** negative staining (100x and 400x magnitude), **B** positive staining (100x and 400x magnitude)

Immunohistochemical staining for activin homodimer subunit inhibin beta A was performed manually as previously described [30]. Briefly, paraffin sections were dewaxed in xylene (2 × 10 minutes at room temperature) and hydrated in a descending alcohol series (5 minutes at room temperature each: 100%/90%/70%/30%/0% ethanol). Epitopes were retrieved heat-induced in a pressure cooker at pH 8 (0.5 M EDTA buffer). After rinsing, sections were incubated with anti-Inhibin beta A from ANSH labs Webster, Texas, US (clone AI006, dilution 1:1500) for 30 minutes at room temperature. For detection, the Dako Universal LSAB2 Kit was used (Agilent). Nuclei were counterstained with hematoxylin (Merck) and slides were coversliped with glycerol gelatin (Merck). Primary antibody was omitted in negative control section. Pictures were acquired via the Vectra 3 Automated Quantitative Pathology Imaging System (Akoya Bioscience) employing Vectra3 (Akoya Bioscience, version 3.0.7). Examples for representative staining are shown in Fig. 1. All cases were scored by two blinded investigators by assessing two cores per tumor sample. Both the proportion of positive tumor cells (< 25%:0 25–50%:1 50–75%:2 > 75%:3) and the staining intensity was assessed (intensity score 1–4, with staining intensity corresponding to: 1: no staining, 2: weak staining 3: moderate staining and 4: strong staining.). Subscores were multiplied to calculate final score from 0 to 12, with a score > 6 interpreted as activin high expression. In the case of disagreement in scoring between the two investigators (JJS and CT), cases were reassessed and discussed. Additionally, staining pattern and scoring was reevaluated and independently confirmed by a pathologist (AA). For a detailed version of the visualizing agents, see Supplemental Table S1.

### Statistics

Statistical analysis was performed using IBM SPSS Version 24. Overall survival was evaluated in months from time of diagnosis until death or until the most recent follow-up using Kaplan–Meier plots. Associations of activin expression with tumor size, distant and lymph node metastasis, venous and lymphatic infiltration, Lauren and Ming classification, grading and UICC classification were tested by the X2 test. Univariate survival analyses were performed according to the Kaplan-Meier method with the log-rank test for assessment of statistical significance. An alpha-level of 0.05 was set prior to all experiments.

## Results

### Clinical characteristics

Data of 277 Patients (detailed clinico-pathological characteristics are summarized in Table 1) were analyzed in this study (female = 100, males = 177, median age = 61.81 years) Patients with all tumor stages (T1 = 38, T2 = 105, T3 = 102, T4 = 31, unknown = 1), nodal and metastasis status (N0 = 71 N + =206, M0 = 196, M1 = 81) were included. Data on lymphatic infiltration was available in 257 cases, data on venous infiltration in 274 cases. Lymphatic infiltration was observed in 165 patients (59.6%), venous infiltration in 90 patients (32.5%). The mean overall survival was 57.25 months (95% CI: 49.23–65.27), the mean disease-specific survival was 70.08 months (95% CI: 60.60–79.56).

### Activin protein expression is common in AEG/ASs and inversely correlated with tumor size, lymph node and distant metastasis

High activin protein expression was observed in 72 out of 277 primary tumors (26.0%). We detected a mixed expression pattern of activin subunit inhibin beta A in tumor cells with predominantly cytoplasmic and marginally nuclear protein expression. Heterogeneity with regards to activin expression scores in between the two TMA cores per tumor sample was very low (difference > 1 point in either intensity or positive tumor cell subscores in 7 out of 277 cases corresponding to 2.4% of cases) Activin protein expression scores were negatively correlated with tumor size, lymph node and distant metastasis (*p* < 0.041, *p* = 0.014 and *p* = 0.003 respectively). Accordingly, we observed a statistically significant inverse correlation of activin expression with UICC stages (see Table 1).

Activin expression score was not significantly correlated with tumor grading (*p* = 0.35). With regard to the Lauren classification, activin expression scores did not differ significantly between tumors of the intestinal, diffuse or mixed type (*p* = 0.11). Higher activin protein expression scores correlated with tumors classified as expansive as opposed to infiltrative following the Ming Classification (*p* = 0.034).

### CD4^+^ T-helper cell tumor invasion correlates with higher activin expression

As immune modulating effects of activin are reported in other tumor entities, we decided to investigate tumor T-cell infiltration as possible confounding factor. We observed high infiltration of T-cells positive for CD3^+^, CD4^+^ and CD8^+^ in 118, 98 and 120 of tumors respectively. (42.6, 35.4, 43.3% of cases). When correlating T-cell infiltration with activin protein expression, we observed no statistically significant correlation of activin expression with CD3^+^ or CD8^+^ T-cells, but a statistically significant correlation with CD4^+^ T-helper-cells (*p* < 0.009). Additionally, we correlated activin with PD-1 and PD-L1 expression in lymphocytes and tumor cells. There was a trend towards higher activin protein expression in tumors with lymphocytes expressing PD-1 and PD-L1 respectively, but this trend did not reach statistical significance (*p* = 0.051 and *p* = 0.065 respectively).

### Activin protein expression is positively correlated with longer overall survival, especially in tumors with high CD4^+^ cell count

**Fig. 2:**
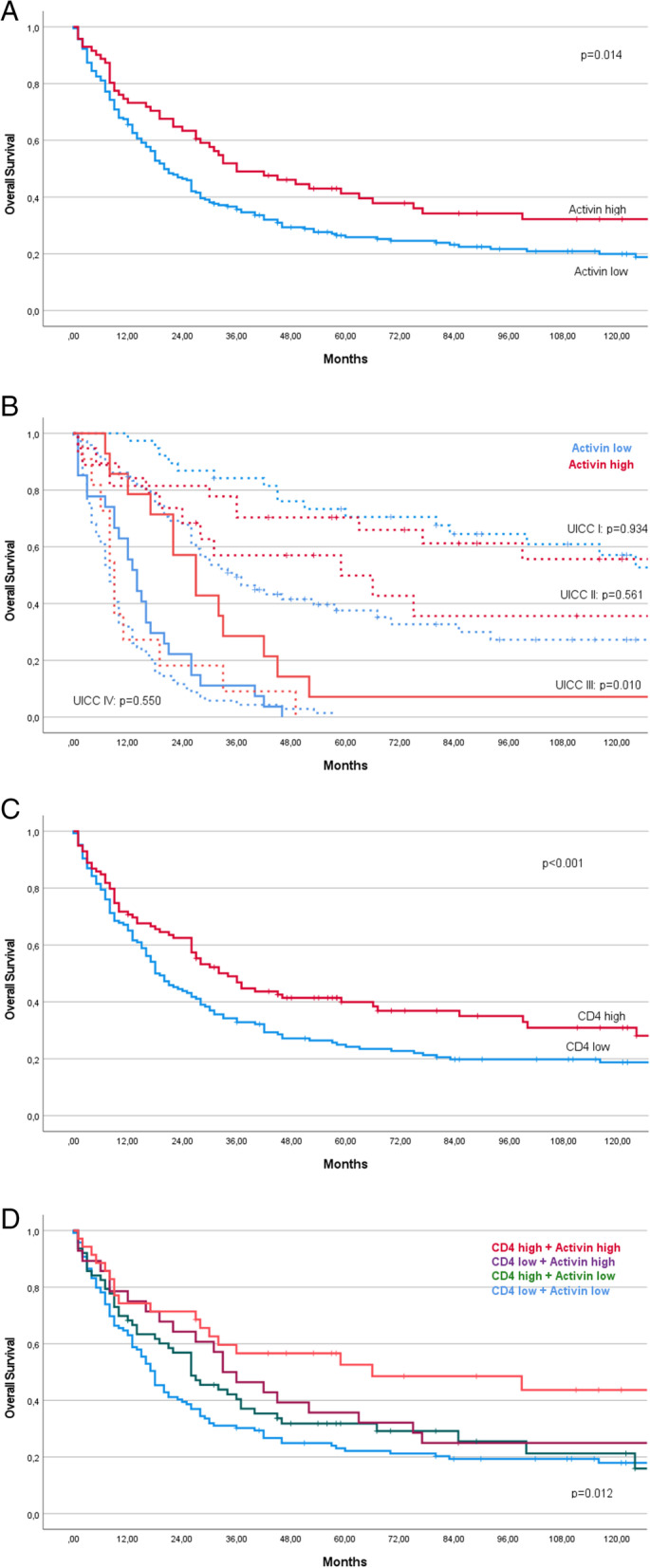
Kaplan-Meier Plots of Survival stratified by Activin expression status (**A**) combined UICC and Activin expression status (**B**) CD4^+^ cell infiltration status (**C**) and for combined Activin and CD4^+^ cell infiltration status (**B**). High Activin expression and high CD4 infiltration are positive prognostic for survival in GC patients. The survival benefit of activin is exclusively seen in UICC III stage (**B**) and in combination with CD4^+^ cell high infiltration status (**D**). Significance calculated by log-rank test

Activin protein expression showed a statistically significant correlation with overall survival (see Fig. 2a) (72.1 +/− 8.27 versus 51.2 +/− 4.6 months, *p* < 0.01). The correlation of activin protein expression and tumor specific survival did not reach statistical significance. When investigating the correlation of activin and overall survival in tumors of different UICC stages, the effect was predominantly found in tumors of UICC stage III (35.5 +/− 9.2 versus 15.6 +/− 2.4 months, p < 0.01) (see Fig. 2b). In a multivariate analysis, only age and UICC stage remained statistical significantly correlated with overall survival (see Table 2).

**Table 2:** Univariate and multivariate survival analyses including age, sex, UICC stage, CD4 positive infiltration and activin state

Clinical Factor		Univariate		Multiple Cox Regression		
	Mean Survival	CI	p	HR	CI	p
Total patients	57.79	49.7–65.8.86				
Age						
≤ 65 years	64.66	53.39–75.92	**0.028**	**1.409**	**1.039–1.911**	**0.027**
> 65 years	47.39	36.86–57.88				
Sex						
Female	60.54	46.93–74.12	0.629	0.838	0.616–1.139	0.258
Male	54.34	44.74–63.94				
UICC stage						
UICC I	113.52	96.68–130.34	< 0.001	**2.332**	**1.985–2.740**	**< 0.001**
UICC II	73.05	57.85–88.26				
UICC III	22.44	14.97–29.91				
UICC IV	11.85	9.23–14.473				
Ming						
Expansive	63.55	51.51–75.69	0.033	1.122	0.814–1.547	0.482
Infiltrative	49.79	39.84–59.74				
CD4				0.988	0.717–1.362	0.943
High	70.34	55.35–85.34	0.017			
Low	49.02	38.97–59.06				
Activin				0.866	0.594–1.262	0.454
High	72.13	55.92–88.34	0.014			
Low	51.28	42.35–60.20				

As published before, CD4^+^ T-cell infiltration is correlated with longer survival in AEG/AS patients (Fig. 2C, [29]). Interestingly, when investigating the correlation of high activin protein expression and CD4^+^ T-cells, we observed improved survival solely in patients with tumors expressing both high activin levels and high CD4^+^ infiltration (median survival 86.8 months +/− 12.1]). Tumors with either exclusively high CD4^+^ cells or exclusively high activin protein expression did not exhibit a longer survival (median survival 55.9 +/− 8.7 and 57.4 +/− 11 months) when compared with tumors low in CD4^+^ cell infiltration and activin expression (median survival 47.1 +/− 5.7 months) (Fig. 2d).

## Discussion

This study presents data on the protein expression of the activin homodimer inhibin beta-A subunit in a comprehensive European cohort of AEG/AS tumors.

Our cohort studied encompasses 277 patients treated at a single center primarily by surgery, we observed high activin homodimer subunit inhibin beta A expression in roughly a quarter of the cases (71/277, 26,0%). Activin expression correlated inversely with tumor stage, and as therefore expected, we observed a statistically significant higher overall survival in patients with tumors exhibiting high activin expression (72.1 +/− 8.3 versus 51.2 +/− 4.6 months, *p* < 0.01). This effect was predominantly found in tumors of UICC stage III (35.5 +/− 9.2 versus 15.6 +/− 2.4 months, p < 0.01). Additionally, higher activin expression scores were seen in tumors of the expansive type rather than tumors of the infiltrative type following the Ming-Classification (*p* = 0.04). A multivariate regression did not show activin subunit protein expression as independent predictor of survival, which is most probably due to the strong inverse correlation of activin with tumor stage in the cohort studied.

These data are complementing prior in vitro data demonstrating a growth inhibiting effect of activin on gastric cancer cell lines [22, 23]. Studies investigating the mRNA expression of activin in AEG/AS cohorts reported a shorter overall survival [25, 27]. This discrepancy in findings could be explained by post transcriptional control of activin levels, as well as genetic differences in the cohorts, as the cohort studied herein is the first predominantly European cohort investigated for activin protein expression in AEG/ASs. Arguably tumor protein activin expression is a more accurate measurement of activin signal activation as compared to activin mRNA. In our cohort, the correlation between overall survival and activin subunit protein expression was most pronounced in UICC stage III cancers. This observation might be due to a stronger pro-proliferative effect through other pathways in these tumors when compared to UICC stage I and II cancers which is mitigated in cases of higher activing protein expression, but further studies are needed. The higher activin expression in tumors of the expansive type rather than tumors of the infiltrative type is most likely due to the correlation of both expansive type [31] as well as activin expression with lower tumor stage.

No correlation was found between activin expression in metastases or lymph nodes and overall survival, albeit a smaller cohort size reduces the power to detect a difference in these cases.

Interestingly, a correlation of activin protein expression and CD4^+^ T- helper cell infiltration was found, but not overall CD3^+^ T-cell infiltration or CD8^+^ cytotoxic T-cell infiltration.

We previously reported on longer survival of patients with AEG/ASs exhibiting high CD4^+^ cell infiltration [29]. Even though a non-statistical significant trend towards correlation between activin protein expression and overall survival was seen in tumors exhibiting a low CD4^+^ T-cell infiltration (high 57.4 +/− 11.0 months versus low 47.1 +/− 5.7 months, p = n.s.), the effect was more pronounced in tumors with high CD4 infiltration score (88.8 +/− 12.1 months versus 55.9 +/− 8.6, *p* < 0.05). Interestingly, the longer overall survival from high CD4^+^ T-cell infiltration and high activin expression were reliant on each other. This finding strongly hints towards a tumor suppressive effect, activin exerts through a yet unspecified effect on CD4^+^ T-cells infiltrating the tumor. Whether this presumed effect is a direct effect on lymphocytes or an indirect effect through activins effect on tumor epithelium or fibroblasts and the induction of other signaling molecules cannot be said at this point. Due to the correlative nature of data presented here, it is also conceivable that combined high activin expression and CD4+ T-cell infiltration a marker for a yet to specify tumor subtype. CD4^+^ T-cells are a heterogeneous group of cells including pro- and anti-inflammatory T-helper cells (Th1 and Th2), IL-17 producing Th17 cells, and regulatory T-cells, and further subclassification of CD4^+^ T-cells should be performed in future studies to possible discern mechanistic interaction between activin and CD4^+^ T-cells in AEG/AS tumors, especially given the recent data on activin driving differentiation towards Th17 cells [21].

Several limitations should be kept in mind when interpreting the data presented here. TMAs are a valuable tool to assay protein expression in large cohorts such as ours, but some concern exists due to the incomplete reflection of tumor heterogeneity, especially with regards to tumor infiltrating lymphocytes. The heterogeneity in between TMA cores, at least for activin staining was low (difference > 1 point in either intensity or positive tumor cell sub-scores in 2,4% of cases), and the tumor infiltrating lymphocyte working group sees TMAs as adequate tool for rapid evaluation of large clinical cohorts [32]. Nevertheless, full reflection of tumor heterogeneity was not possible due to our approach, a limitation that should be kept in mind. In our study, we investigated protein expression of the activin homodimer subunit inhibin beta A. Inhibin beta A dimerizes with either the inhibin alpha subunit or Inhibin beta subunit, for which several isoforms exist. A cross-reactivity with other homo- and heterodimers of inhibin beta A besides activin A cannot be ruled out by immunohistochemistry, an important limitation when interpreting the results of this study.

In conclusion, the data presented herein show a correlation of higher activin tumor protein expression and longer overall survival in a cohort of patients with AEG/AS tumors. The data imply a net anti-tumorigenic effect of activin signaling in this tumor entity, albeit due to the context specific nature of activin signaling observed in other tumor entities a more distinguished function with a net pro tumorigenic effect in a subset of tumors is conceivable. Therefore, further mechanistic in vitro and in vivo studies of activin in the context of AEG/ASs, especially with regards to its effect on CD4^+^ tumor infiltrating lymphocytes, are warranted.

## Supplementary Information

**Additional file 1: Supplemental Table S1.** Overview of visualized antigens and visualizing agent.

**Additional file 2: Figure S1.** Distribution of activin high and activin low expressing tumors by tumor stage illustrating the inverse correlation of activin with UICC tumor stage.

## Data Availability

All data generated or analyzed during this study are included in this published article and its supplementary information files.

## Abbreviations

UICC: Union for International Cancer Control
AEG: Adenocarcinomas of the esophagus
AS: Adenocarcinomas of the stomach
TGF-β: Transforming growth factor-beta
TMA: Tissue microarray

## References

[1] Sung H (2021). Global Cancer statistics 2020: GLOBOCAN estimates of incidence and mortality worldwide for 36 cancers in 185 countries. CA Cancer J Clin.

[2] Hooi JKY (2017). Global prevalence of helicobacter pylori infection: systematic review and Meta-analysis. Gastroenterology.

[3] Recio-Boiles A, Babiker HM (2021). Esophageal Cancer.

[4] Jung B, Staudacher JJ, Beauchamp D (2017). Transforming growth factor beta superfamily signaling in development of colorectal Cancer. Gastroenterology.

[5] Phillips DJ (2005). Activin a: from sometime reproductive factor to genuine cytokine. Vet Immunol Immunopathol.

[6] Jones KL (2000). Activin a release into the circulation is an early event in systemic inflammation and precedes the release of follistatin. Endocrinology.

[7] Jones KL (2007). Activin a is a critical component of the inflammatory response, and its binding protein, follistatin, reduces mortality in endotoxemia. Proc Natl Acad Sci U S A.

[8] Phillips DJ (2001). Evidence for activin a and follistatin involvement in the systemic inflammatory response. Mol Cell Endocrinol.

[9] Staudacher JJ (2017). Activin in acute pancreatitis: potential risk-stratifying marker and novel therapeutic target. Sci Rep.

[10] Chen YG (2006). Activin signaling and its role in regulation of cell proliferation, apoptosis, and carcinogenesis. Exp Biol Med.

[11] Togashi Y (2015). Activin signal promotes cancer progression and is involved in cachexia in a subset of pancreatic cancer. Cancer Lett.

[12] Chen JL (2014). Elevated expression of activins promotes muscle wasting and cachexia. FASEB J.

[13] Willis SA (1996). Formation and activation by phosphorylation of activin receptor complexes. Mol Endocrinol.

[14] Bauer J (2015). Activin and TGFbeta use diverging mitogenic signaling in advanced colon cancer. Mol Cancer.

[15] Jana A (2017). NFkB is essential for activin-induced colorectal cancer migration via upregulation of PI3K-MDM2 pathway. Oncotarget.

[16] Loomans HA, Andl CD (2014). Intertwining of Activin a and TGFbeta signaling: dual roles in Cancer progression and Cancer cell invasion. Cancers (Basel).

[17] Sanjabi S (2009). Anti-inflammatory and pro-inflammatory roles of TGF-beta, IL-10, and IL-22 in immunity and autoimmunity. Curr Opin Pharmacol.

[18] Sideras P (2013). Activin, neutrophils, and inflammation: just coincidence?. Semin Immunopathol.

[19] Jones CP (2012). Activin a and TGF-beta promote T(H)9 cell-mediated pulmonary allergic pathology. J Allergy Clin Immunol.

[20] De Martino M (2021). Activin a promotes regulatory T-cell-mediated immunosuppression in irradiated breast Cancer. Cancer Immunol Res.

[21] Wu B (2021). The TGF-beta superfamily cytokine Activin-a is induced during autoimmune neuroinflammation and drives pathogenic Th17 cell differentiation. Immunity.

[22] Kaneda H (2011). Activin a inhibits vascular endothelial cell growth and suppresses tumour angiogenesis in gastric cancer. Br J Cancer.

[23] Kim YI (2009). Cell growth regulation through apoptosis by activin in human gastric cancer SNU-16 cell lines. Oncol Rep.

[24] Chen ZL (2019). INHBA gene silencing inhibits gastric cancer cell migration and invasion by impeding activation of the TGF-beta signaling pathway. J Cell Physiol.

[25] Oshima T (2014). Relation of INHBA gene expression to outcomes in gastric cancer after curative surgery. Anticancer Res.

[26] Katayama Y (2017). Clinical significance of INHBA gene expression in patients with gastric Cancer who receive curative resection followed by adjuvant S-1 chemotherapy. In Vivo.

[27] Wang Q (2012). Upregulated INHBA expression is associated with poor survival in gastric cancer. Med Oncol.

[28] Semitekolou M (2009). Activin-a induces regulatory T cells that suppress T helper cell immune responses and protect from allergic airway disease. J Exp Med.

[29] Potzsch M (2020). Better prognosis of gastric cancer patients with high levels of tumor infiltrating lymphocytes is counteracted by PD-1 expression. Oncoimmunology.

[30] Staudacher JJ (2017). Activin signaling is an essential component of the TGF-beta induced pro-metastatic phenotype in colorectal cancer. Sci Rep.

[31] Luebke T (2005). Histological grading in gastric cancer by Ming classification: correlation with histopathological subtypes, metastasis, and prognosis. World J Surg.

[32] Salgado R (2015). The evaluation of tumor-infiltrating lymphocytes (TILs) in breast cancer: recommendations by an international TILs working group 2014. Ann Oncol.

