# Supplementary material for: Prognostic impact of activin subunit inhibin beta A in gastric and esophageal adenocarcinomas
Source: BMC Cancer. 2022 Sep 5;22:953. doi: 10.1186/s12885-022-10016-5 (PMC9446826; doi:10.1186/s12885-022-10016-5)
Supplement: Supplementary file 1 — Additional file 1: Supplemental Table S1. Overview of visualized antigens and visualizing agent. [file 12885_2022_10016_MOESM1_ESM.docx]

| Visualized Antigen | Antibody | Order number | Manufacturer |
| --- | --- | --- | --- |
| CD3 | LN10 | NCL-L-CD3-565 | Novocastra^®^ |
| CD4 | 4B12 | CD4-368-L-C | Novocastra^®^ |
| CD8 | C8/144B | M7103 | DAKO^®^ |
| PD-L1 | E1L3N | - | Cell Signaling technologies^®^ |
| PD-1 | EP239 | - | Epitomics^®^ |
| Activin | AB-305-AI006 |  | ANSH labs^®^ |

*Supplemental table S1: Overview of visualized antigens and visualizing agent.*
