# Supplementary material for: Prognostic impact of activin subunit inhibin beta A in gastric and esophageal adenocarcinomas
Source: BMC Cancer. 2022 Sep 5;22:953. doi: 10.1186/s12885-022-10016-5 (PMC9446826; doi:10.1186/s12885-022-10016-5)
Supplement: Supplementary file 2 — Additional file 2: Figure S1. Distribution of activin high and activin low expressing tumors by tumor stage illustrating the inverse correlation of activin with UICC tumor stage. [file 12885_2022_10016_MOESM2_ESM.docx]

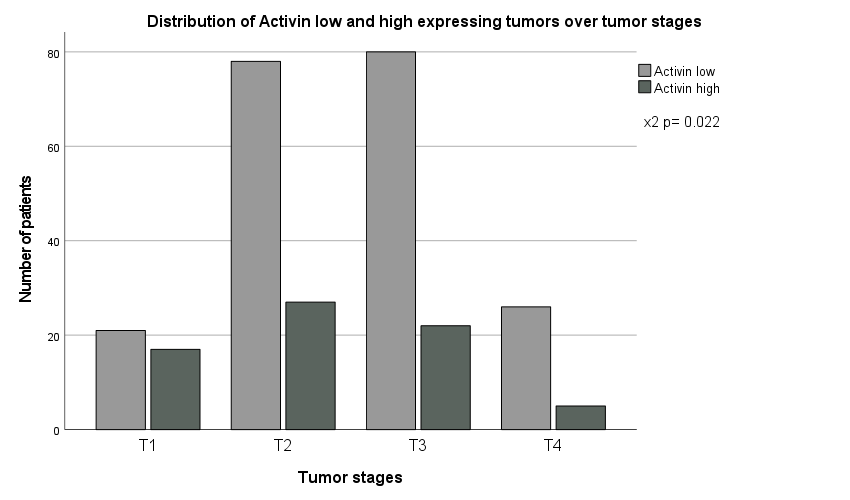
*Figure S1: Distribution of activin high and activin low expressing tumors by tumor stage illustrating the inverse correlation of activin with UICC tumor stage*
